# Communication Link Analysis of a Low-Earth Orbit Satellites Considering Interference Sources Moving Along Various Parabola-Curved Paths

**DOI:** 10.3390/s24248185

**Published:** 2024-12-21

**Authors:** Hyunmu Kang, Eunjung Kang, Hosung Choo

**Affiliations:** 1 Department of Electronic and Electrical Engineering, Hongik University, Seoul 04066, Republic of Korea; mumu0908@mail.hongik.ac.kr (H.K.); hschoo@hongik.ac.kr (H.C.); 2 Missile Research Institute, Agency for Defense Development, Daejeon 34186, Republic of Korea

**Keywords:** LEO satellite, communication link, interference situation, airborne interference source path, *J*/*S* ratio

## Abstract

We analyze the communication link of an LEO satellite considering interference sources moving along various parabola-curved paths. In this situation, the location of the ground station, airborne interference source paths, and the satellite’s trajectory were expressed in the East-North-Up (ENU) coordinate system. The airborne interference source path is designed using a parabola equation with a directrix parallel to the satellite’s trajectory to analyze the interference situation for more diverse interference source paths, rather than using a straight path. To investigate critical interference situations where the *J*/*S* ratio is maintained above −20 dB with a small deviation during the communication time, we investigate interference situations by changing the parameters of the interference source path. The genetic algorithm (GA) is used to easily find an airborne interference source path that maintains the *J*/*S* ratio above −20 dB with a small deviation. A cost function for the GA is then defined as the average difference between the *J*/*S* ratio and the reference value (−10 dB and −20 dB) during the communication time. The optimum parameters of the interference source path are determined at a minimum cost in the GA. These results demonstrate that more significant interference situations for the communication link can be easily found by using parabola-curved paths and the GA. As a result, previous studies investigated the basic tendency of the *J*/*S* ratio using straight paths. However, this study provides a database for operating an anti-jamming system based on the obtained optimized path.

## 1. Introduction

In recent decades, LEO satellites have been increasingly used for precise Earth observations, including military reconnaissance, terrain monitoring, and climate changes. These LEO satellites often employ a synthetic aperture radar (SAR) to capture high-resolution images of the Earth’s surface, and then transmit them to ground stations for all-weather monitoring [1,2,3,4,5,6]. They move along their own orbits at altitudes ranging from 250 km to 2000 km at high speeds and transmit the high-resolution image data to ground stations through a downlink within a communication time of around 10 min [7,8,9]. To receive the data at the ground station during the limited communication time, it is necessary to perform the communication link analysis considering various interference situations. In communication links, in particular, the impact of electromagnetic (EM) interference sources near ground stations affects the communication link more significantly than interference factors such as losses, attenuation, and Doppler shift in the Earth’s environment [10,11,12,13,14,15,16,17]. Therefore, it is required to conduct communication link analysis considering EM interference sources in various scenarios. Previous studies have analyzed scenarios where the interference source is fixed at a specific location [18,19,20,21]. However, for the airborne interference source, if the power ratio of the interference and the satellite signal received by the ground station remains consistent, it is difficult for the ground station to distinguish between the two. Although there have been cases in which the situation of a moving interference source has been investigated [22,23,24,25], only interference situations with a simple straight path have been analyzed. Therefore, it is essential to further investigate using communication link analysis for interference situations in which there is a small deviation in the power ratio between the interference signal and the satellite signal at the ground station.

In this paper, we analyze the communication link of an LEO satellite, considering interference sources moving along various parabola-curved paths. To analyze communication links for more various and practical interference source paths, we employ parabola equations for curved paths. This parabola equation can express both curved and straight paths, facilitating link analysis for various interference source paths. The genetic algorithm (GA) is used to find the paths that cause more significant interference situations where the power ratio (i.e., the jamming-to-signal (*J*/*S*) ratio) between the interference signals and the satellite signals has a minimum deviation. A cost function in the GA is defined as the average difference between the *J*/*S* ratio and the reference value (−10 dB and −20 dB) during the communication time. We then compare the *J*/*S* ratio results between the optimized path (for −10 dB and −20 dB references) and the straight path. These results demonstrate that more significant interference situations for the communication link can be easily found by using parabola-curved paths and the GA. To explain the analysis results of LEO satellite communication links considering airborne interference sources, this paper is organized as follows. Chapter 2 introduces the analysis of LEO satellite communication links under interference situations. In Chapter 3, we investigate the interference situation using the GA. Finally, Chapter 4 summarizes the results of this study.

## 2. Analysis of LEO Satellite Communication Links Under Interference Situations

### 2.1. Communication Link Considering Parabola-Curved Paths of Airborne Interference Source

**Figure 1:**
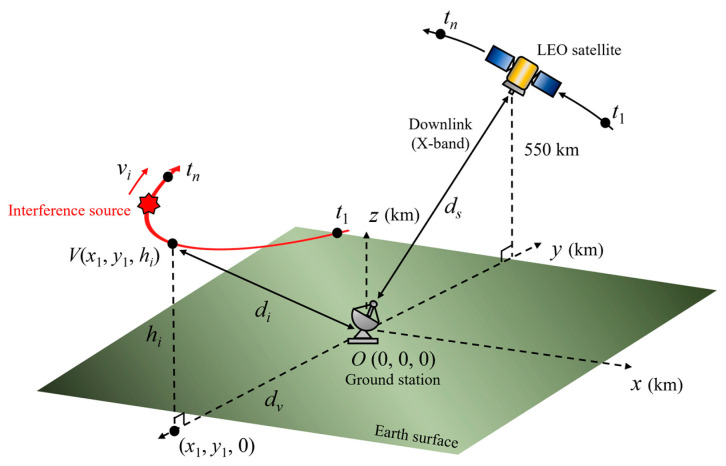
LEO satellite communication link under the interference situation based on the ENU coordinate system.

Figure 1 shows the LEO satellite communication link under the interference scenario when the LEO satellite moves along the trajectory at an altitude of 550 km and transmits the image data to the ground station. Because the time for the LEO satellite to transmit the image data to the ground station is limited to about 10 min, the data communication time from *t*_1_ to *t_n_* is set to 600 s. In this situation, the location of the ground station, the airborne interference source path, and the satellite’s trajectory are expressed in the East-North-Up (ENU) coordinate system, which uses Cartesian coordinates to represent a specific location relative to the local origin. The ground station is the local origin of *O*(0, 0, 0), and the airborne interference source moves at a velocity *v_i_* along the red parabola curved path with an altitude *h_i_*. *d_i_* is the slant distance between the interference source and the ground station. *d_s_* is the slant distance between the LEO satellite and the ground station. *V*(*x*_1_, *y*_1_, *h_i_*) is the vertex of the parabola curved path, and *d_v_* is the distance between the (*x*_1_, *y*_1_, 0) and the ground station.

**Figure 2:**
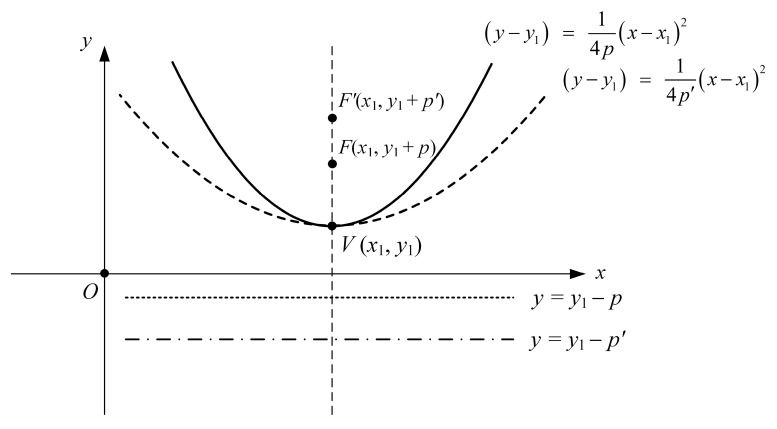
Parabola equations for the airborne interference source path based on the ENU coordinate system.

To analyze communication links for more various and practical interference source paths, we use parabola equations to express the airborne interference source paths rather than using a simple straight path. Figure 2 illustrates the parabola equations for the airborne interference source path, which is represented based on the ENU coordinate system. When the interference source is located at an altitude *h_i_*, the interference source path on the *xy*-plane (*z* = *h_i_*) is expressed as Equation (1).

(1) $$\left(y-y_{1}\right)=\frac{1}{4p}{\left(x-x_{1}\right)}^{2},$$

*F*(*x*_1_, *y*_1_ + *p*) represents the focal point of the parabola equation, and *V*(*x*_1_, *y*_1_) is the vertex of the parabola equation. As the value of *p* increases, the parabola curved path is similar in form to a straight path. For example, if *p* increases to *p*’, then the parabola path becomes closer to the straight *x*-axis line. This parabola equation, which includes both curved and straight paths, facilitates the investigation of a variety of interference source paths.

### 2.2. Analysis of Communication Links According to Various Paths of the Airborne Interference Source

**Figure 3:**
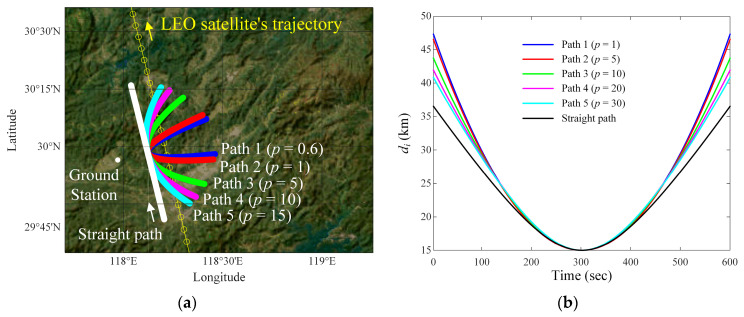

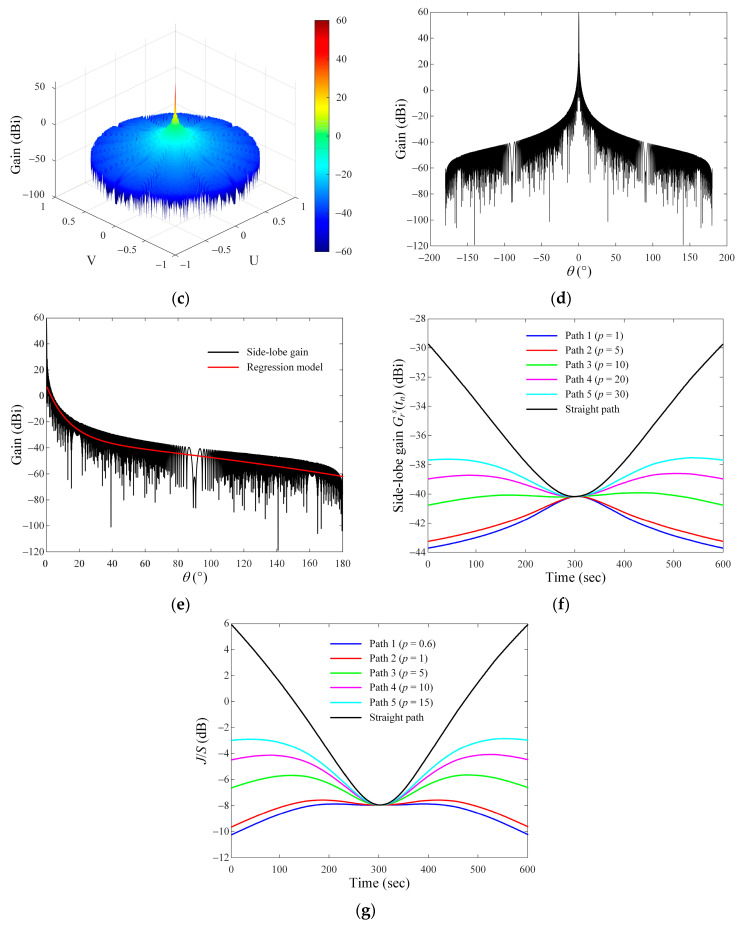
The LEO satellite communication link analysis for the parabola-curved and straight paths of the airborne interference source according to the variation in p (when *d_v_* = 15 km, *h_i_* = 9 km, and *v_i_* = 400 km/h): (**a**) parabola-curved and straight paths of the interference source on geodetic coordinates map; (**b**) *d_i_* of the interference source at an altitude of *h_i_*; (**c**) 3D radiation pattern of the ground station antenna in UV domain; (**d**) 2D radiation pattern of the ground station antenna; (**e**) side-lobe gain of the ground station antenna with the regression model; (**f**) side-lobe gain of the ground station antenna according to airborne interference paths; (**g**) results of the *J*/*S* ratio according to focal point *p*.

To find an airborne interference source path that maintains the *J*/*S* ratio above −20 dB with a small deviation during the communication time, we investigate interference situations by changing the distance of *d_v_*, altitude of *h_i_*, and the velocity of *v_i_*. Figure 3 shows the LEO satellite communication link analysis for the parabola curved and straight paths of the airborne interference source according to the change in *p*. Figure 3a illustrates the ground station, the LEO satellite’s trajectory, and the airborne interference source paths. The ground station is located at 29.94° latitude and 117.97° longitude. The LEO satellite’s trajectory is obtained from the two-line element (TLE) data provided by the Joint Space Operations Center (JSpOC) at Vandenberg Air Force Base. The TLE data contains the information pertaining to the LEO satellite’s location [26]. LEO satellites can communicate with the ground station when their elevation angle is between 10° and 170°, and have communication coverage within this range [27,28]. To consider the communication coverage, we used TLE data with an elevation angle between 10° and 170° as the satellite trajectory. The LEO satellite moves along a trajectory and communicates with the ground station at a fixed location during a limited time from *t*_1_ to *t_n_*. The airborne interference source moves along the parabola curved and straight paths at an altitude of *h_i_* = 9 km with a velocity *v_i_* = 400 km/h. These parabola-curved paths have a directrix that is parallel to the LEO satellite’s trajectory, and *d_v_* is 15 km. We set the parabola-curved paths according to the focal point as Path 1 (*p* = 0.6), Path 2 (*p* = 1), Path 3 (*p* = 5), Path 4 (*p* = 10), and Path 5 (*p* = 15). These *J*/*S* ratios of parabola-curved paths are then compared with that of the straight path, where the straight path is set parallel to the satellite’s trajectory. Figure 3b shows the slant distance between the interference source (at an altitude of *h_i_* = 9 km) and the ground station based on the ENU coordinates system, where the solid lines indicate the slant distance *d_i_*. The maximum and minimum values of the *d_i_* are compared for each interference source path and listed in Table 1.

Figure 3c shows the 3D radiation pattern of a parabolic reflector antenna used in the ground station in the UV domain. The ground station antenna pattern is theoretically calculated using MATLAB R2022b based on geometrical optics (GO) and physical optics (PO) methods [29,30,31]. The diameter of the parabolic reflector is 11.3 m, and a rectangular horn antenna is used for a feed antenna. At 8 GHz, it has a bore-sight gain of 59 dBi, and a half-power beamwidth of 0.2°. In the LEO satellite communication system, the ground station antenna receives the image data by tracking accurately at the bore-sight direction of the satellite. Therefore, the side-lobe gain of the ground station antenna for the direction where the ground station is exposed to interference signals varies with time. Figure 3d,e shows overall 2D radiation patterns and side-lobe gain with the regression model of the ground station antenna. We derived the side-lobe gain and relative angle difference *ψ* using the same method as our previous study [25] and applied it in this study. To easily observe the tendency of the *J*/*S* ratio, we used the regression model (red line), as shown in Figure 3e. Figure 3f represents the side-lobe gain of the ground station antenna to the direction from which the interference signals are incoming during the communication time according to the interference source paths. The case of the straight path is illustrated as the black solid line, and those of the parabola paths (Paths 1 to 5) are represented by solid lines in blue, red, green, magenta, and cyan, respectively. The average side-lobe gains for each parabola curved path are represented as −42.2 dBi, −41.8 dBi, −40.2 dBi, −39.2 dBi, and −38.5 dBi, respectively. For the straight path, it is observed as −35.6 dBi. To analyze the communication link of the LEO satellite for various parabola-curved paths, the *J*/*S* ratio is calculated using Equations (2) and (3),

(2) $$\frac{J(t_{n})}{S(t_{n})}=\frac{P_{t}^{i}+G_{t}^{i}+G_{r}^{s}(t_{n})-L_{i}(t_{n})}{P_{t}^{s}+G_{t}^{s}+G_{r}^{g}-L_{s}(t_{n})},$$

(3) $$L_{i}(t_{n})=20\log_{10}(\sqrt{{\left(\sqrt{4p(y(t_{n})-y_{1})}+x_{1}\right)}^{2}+{\left(y(t_{n})\right)}^{2}+h_{i}^{2}})+20\log_{10}(f)-147.55,$$

(4) $$L_{s}\left(t_{n}\right)=20\log_{10}\left(d_{s}\left(t_{n}\right)\right)+20\log_{10}\left(f\right)-147.55,$$

where $S(t_{n})$ is the received power at the ground station, and $P_{t}^{s}$ is the transmission power of the LEO satellite. $G_{t}^{s}$ is the bore-sight gain of the satellite antenna, and $G_{r}^{g}$ is the bore-sight gain of the ground station antenna. $L_{s}(t_{n})$ is the path loss from the satellite to the ground station and is calculated using Equation (4). $J(t_{n})$ is the received power at the ground station from the interference source that moves along a parabola curved path. $P_{t}^{i}$ is the transmission power of the interference source, and $G_{t}^{i}$ is the bore-sight gain of the interference source antenna. $G_{r}^{s}(t_{n})$ is the side-lobe gain of the ground station antenna for the direction where the ground station is exposed to interference signals according to the time. $L_{i}(t_{n})$ is the path loss from the interference source to the ground station and is calculated using Equation (3) [32,33]. Detailed parameters for the LEO satellite communication link simulation considering the interference situation are listed in Table 2.

Figure 3g shows the resulting *J*/*S* ratios according to the focal point *p*. The average *J*/*S* ratios for the parabola paths are −8.5 dB (Path 1), −8.1 dB (Path 2), −6.4 dB (Path 3), −5.3 dB (Path 4), and −4.6 dB (Path 5), respectively. For the straight path, the average and minimum of the *J*/*S* ratios are −1.3 dB and −7.9 dB. We then observe the average difference between the *J*/*S* ratio of each interference source path and the value (=−7.9 dB) when the satellite is closest (*t* = *t*_300_) to the ground station during the communication time. For interference source paths, the average differences are 0.6 dB (Path 1), 0.4 dB (Path 2), 1.5 dB (Path 3), 2.6 dB (Path 4), and 3.3 dB (Path 5), respectively. The average difference for the straight path is observed to be 6.7 dB. When the airborne interference source moves along Path 2, the *J*/*S* ratio is maintained above −20 dB with the smallest deviation. Detailed values are listed in Table 3.

**Figure 4:**
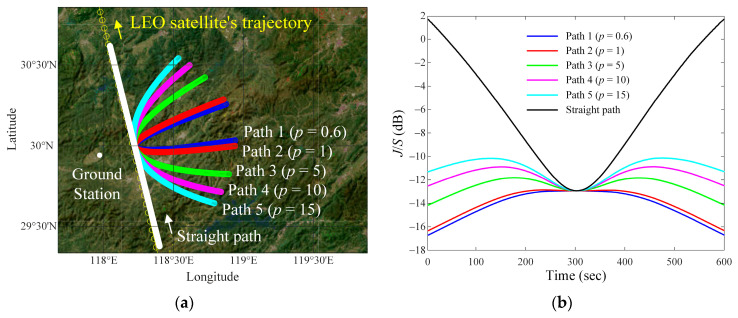
The LEO satellite communication link analysis for the other case of interference situations with various airborne interference source paths, according to *p* (when *d_v_* = 25 km, *h_i_* = 12 km, and *v_i_* = 850 km/h): (**a**) parabola curved and straight paths of the interference source on geodetic coordinates map; (**b**) results of *J*/*S* ratios according to *p*.

Figure 4a presents the LEO satellite communication link analysis for the other case of interference situations according to *p*, where *d_v_* = 25 km, *h_i_* = 12 km, and *v_i_* = 850 km/h. Similarly to the previous case, these parabola-curved paths have a directrix that is parallel to the LEO satellite’s trajectory. Figure 4b shows the resulting *J*/*S* ratios according to *p*, and they are Path 1 (*p* = 0.6), Path 2 (*p* = 1), Path 3 (*p* = 5), Path 4 (*p* = 10), Path 5 (*p* = 15), and the straight path. The parabola-curved paths and the straight path are indicated by blue, red, green, magenta, cyan, and white lines, respectively. The average *J*/*S* ratios for the parabola paths are −14.1 dB (Path 1), −13.9 dB (Path 2), −12.5 dB (Path 3), −11.6 dB (Path 4), and −11 dB (Path 5), respectively. For the straight path, the average *J*/*S* ratio is −5.9 dB. We again observe the average difference between the *J*/*S* ratio of each interference source path and the value (= −12.9 dB) when the satellite is closest (*t* = *t*_300_) to the ground station. The average differences are 1.2 dB (Path 1), 1 dB (Path 2), 0.6 dB (Path 3), 1.3 dB (Path 4), 1.9 dB (Path 5), and 7 dB (straight path), respectively. For Path 3, the *J*/*S* ratio is maintained above −20 dB with the smallest deviation, as listed in Table 4.

## 3. Investigation of the Interference Situation Using the Genetic Algorithm

**Figure 5:**
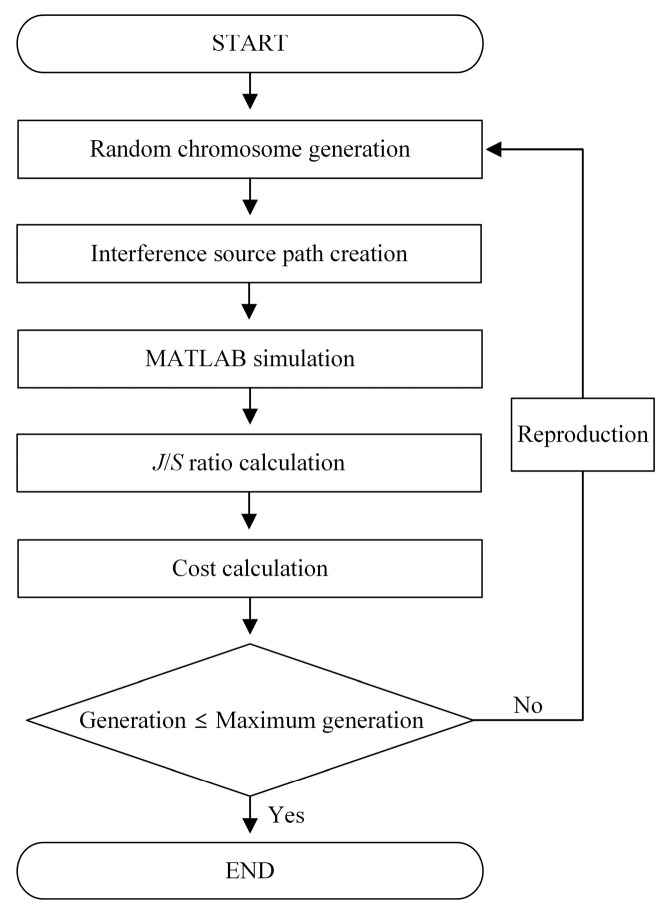
Flowchart of the GA to find the airborne interference source paths that have the smallest deviation in the *J*/*S* ratio.

The GA is used to find the paths that cause more significant interference situations where the *J*/*S* ratio is maintained above −20 dB with a minimum deviation. Herein, GA has the advantage of not being limited to local optimization, unlike other conventional optimization methods, due to crossover and mutation. Therefore, GA is adopted in this study, which requires optimization for multiple parameters of *p*, *d_v_*, *v_i_*, and *h_i_*. Figure 5 represents the flowchart of the GA to find the optimum parameters (*p*, *d_v_*, *v_i_*, and *h_i_*) of the airborne interference source path that have the smallest deviation in the *J*/*S* ratio. The initial parameters are set within the ranges of *p* from 0.1 to 450, *d_v_* from 14 to 65 km, *v_i_* from 400 to 850 km/h, and *h_i_* from 9 to 15 km, respectively. The initial parameters are generated as a uniform distribution within those ranges. First, to find the optimum parameters of the interference source path, a random chromosome is generated, and the decoded values from the chromosome are applied to the parameters of the interference source path. The parameters are then used as the input data to MATLAB R2022b to calculate the *J*/*S* ratio during the communication time. The cost function for the GA is defined as follows:

(5) $$Cost=\frac{1}{n}\sum_{t=1}^{n}\sqrt{{\left(JSR_{t}-JSR_{ref}\right)}^{2}},(t=1,t=2,\ldots ,t=600),$$

where *n* is the total communication time of 600 s, and *JSR_t_* represents the respective *J*/*S* ratio during the time from *t* = 1 to *t* = 600. *JSR_ref_* is set to −10 dB or −20 dB as a reference value of the *J*/*S* ratio. *Cost* is then defined as the average difference between the *J*/*S* ratio and the reference value *JSR_ref_* during the communication time. In the GA process, the parameters of the interference source path are optimized to minimize the *Cost* through the reproduction. Consequently, the optimum parameters of the interference source path are determined at a minimum *Cost* in the GA. In this study, the GA process is repeated for 15 generations with a population of 50, a crossover ratio of 40%, and a mutation ratio of 25%. The MATLAB R2022b simulations have been conducted on a computer with an Intel Core i5-12500 CPU and 128 GB of RAM.

**Figure 6:**
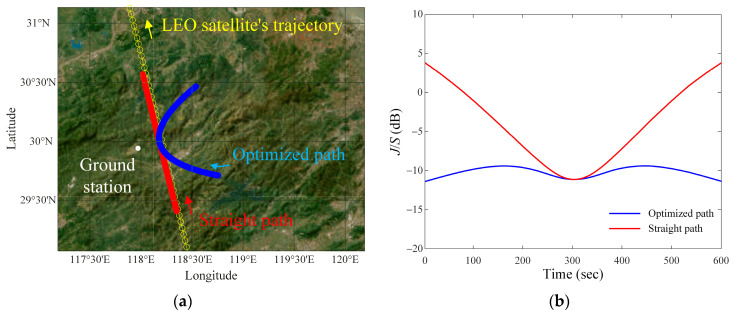
Optimized and straight paths of the airborne interference source when *JSR_ref_* = −10 dB: (**a**) optimized and straight paths on geodetic coordinates map; (**b**) results of the *J*/*S* ratio of the optimized and straight paths.

Figure 6a shows the results of the optimized path that has the smallest average difference between the *J*/*S* ratio and the reference value (*JSR_ref_* = −10 dB) compared with that of the straight path. We choose two paths to compare the *J*/*S* ratio results between the normal path (straight path) and the optimized curved path. The straight path is generally used for the interference path during the communication time, and the curved path is obtained through optimization to maintain the *J*/*S* ratio constant during the communication time. The optimized and straight paths are indicated by blue and red solid lines, respectively. The parameters for the optimized path are *p* = 10.1, *d_v_* = 21.1 km, *v_i_* = 797.4 km/h, and *h_i_* = 11.6 km, respectively. The parameters for the straight path are *p* = 450, *d_v_* = 21.1 km, *v_i_* = 797.4 km/h, and *h_i_* = 11.6 km. Figure 6b shows the *J*/*S* ratio results observed when the interference source moves along the optimized path (blue line) and straight path (red line). The average difference between the *J*/*S* ratio of the optimized path and the *JSR_ref_* (=−10 dB) is observed to be 0.5 dB. The average difference for the straight path is 6.2 dB.

**Figure 7:**
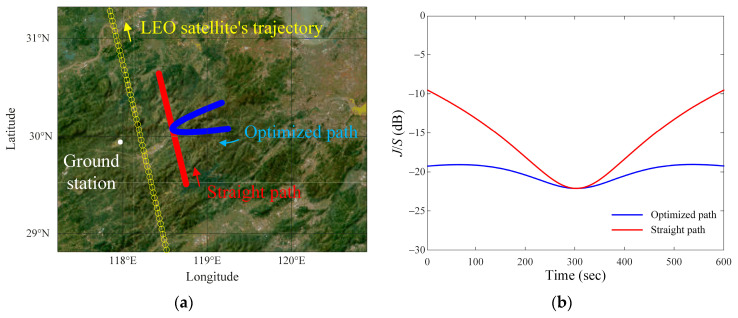
Optimized and straight paths when *JSR_ref_* = −20 dB: (**a**) optimized and straight paths on geodetic coordinates map; (**b**) results of the *J*/*S* ratio of the optimized and straight paths.

We again investigate the path that has the smallest average difference with a reference value of −20 dB. The settings in the GA are used similarly, but only *JSR_ref_* is changed to −20 dB. Figure 7a shows the optimized path, the straight path, the ground station, and the LEO satellite’s trajectory. The optimized parameters for the airborne interference source path are then *p* = 0.9, *d_v_* = 61.5 km, *v_i_* = 775.7 km/h, and *h_i_* = 14.7 km, respectively. The parameters for the straight path are then *p* = 450, *d_v_* = 61.5 km, *v_i_* = 775.7 km/h, and *h_i_* = 14.7 km. Figure 7b shows the resulting *J*/*S* ratios for the optimized and the straight paths. The average difference between the *J*/*S* ratio of the optimized path and the *JSR_ref_* (=−20 dB) is 0.9 dB. For the straight path, the average difference is observed to be 4.8 dB.

To effectively use our method, we summarize the process of obtaining the results of this paper into a five-step protocol.

Detailed procedure of this method:

1. Setting LEO satellite trajectories and the location of the ground station.
  − Loading the information of LEO satellite trajectories from the JSpOC at Vandenberg Air Force Base (TLE data).
  − Specifying the location of the ground station that communicates with the LEO satellite.
2. Expression of interference source paths.
  − Expressing airborne interference source paths based on the parabola equations.
  − Considering conditions such as speed and altitude of actually operable airborne interference sources.
3. Calculation of the *J*/*S* ratio.
  − Calculating the power ratio of the interference and the satellite signal received by the ground station.
  − Considering LEO satellites trajectories and airborne interference source paths.
4. Optimization of airborne interference source paths.
  − Setting the key parameters that determine the interference source path as GA parameters.
  − Setting the Cost to obtain a path with a constant J/S ratio during communication time using GA.
5. Analysis of LEO satellite communications links.
  − Predicting the interference source paths that could seriously affect LEO satellite communications.
  − Providing a database for operating an anti-jamming system based on the obtained optimized path.

We aim to contribute to establishing the principles for analyzing LEO satellite communication links in the presence of airborne interference. It can provide a useful database for operating an anti-jamming system by predicting the interference source path that has a serious impact on LEO satellite communication links. Therefore, the analysis results are expected to contribute to constructing an anti-jamming environment for LEO communication links based on various interference source paths in the future.

## 4. Conclusions

In this paper, we investigated the communication link of the LEO satellite, considering interference sources moving along various parabola-curved paths. We employed parabola equations to express both curved and straight paths. Accordingly, we analyzed communication links for more various and practical interference source paths using parabola-curved paths. The GA was also used to find the paths that cause more significant interference situations where the *J*/*S* ratio is maintained above −20 dB with a minimum difference. Through the link analysis with the GA, the average difference *J*/*S* ratio of the optimized path (for −10 dB and −20 dB references) was found to be 5.7 dB and 3.9 dB smaller compared to the straight path, respectively. These results demonstrated that more significant interference situations for the communication link can be easily found by using parabola-curved paths and the GA. Based on this study, our future work will focus on analyzing more complex LEO satellite communication link scenarios where two or more interference sources are in operation.

## Figures and Tables

**Table 1:** The maximum and minimum values of *d_i_* according to the interference source paths.

Interference Source Paths	Values	
	Max. Value of *d_i_* (km)	Min. Value of *d_i_* (km)
Path 1 (*p* = 0.6)	47.3	15
Path 2 (*p* = 1)	46.6	15
Path 3 (*p* = 5)	43.8	15
Path 4 (*p* = 10)	42	15
Path 5 (*p* = 15)	40.9	15
Straight path	36.6	15

**Table 2:** Parameters for the LEO satellite communication link simulation considering the interference situation.

Parameters	Values
Frequency f (GHz)	8.025
Bore-sight gain $G_{r}^{g}$ (dBi)	59
LEO satellite altitude (km)	550
$\mathrm{Transmission}\;\mathrm{power}\;P_{t}^{s}$ (dBm)	30
Bore-sight gain $G_{t}^{s}$ (dBi)	4.4
Free-space path loss (dB)	$L_{s}(t_{n})$ $\mathrm{and}\;L_{i}(t_{n})$
Interference source velocity *v_i_* (km/h)	400~850
Interference source altitude *h_i_* (km)	9~15
$\mathrm{Transmission}\;\mathrm{power}\;P_{t}^{i}$ (dBm)	70
Bore-sight gain $G_{t}^{i}$ (dBi)	30

**Table 3:** The max/min values and average differences in the *J*/*S* ratio according to the interference source paths (when *d_v_* = 15 km, *h_i_* = 9 km, and *v_i_* = 400 km/h).

Interference Source Paths	*J*/*S* Ratios (dB)		
	Max. Value	Min. Value	Average Difference
Path 1 (*p* = 1)	−7.9	−10.2	0.6
Path 2 (*p* = 5)	−7.6	−9.6	0.4
Path 3 (*p* = 10)	−5.6	−7.9	1.5
Path 4 (*p* = 20)	−4.1	−7.9	2.6
Path 5 (*p* = 30)	−2.8	−7.9	3.3
Straight path	5.9	−7.9	6.7

**Table 4:** The max/min values and average differences in the *J*/*S* ratio according to the interference source paths (when *d_v_* = 25 km, *h_i_* = 12 km, and *v_i_* = 850 km/h).

Interference Source Paths	Values		
	Max. Value	Min. Value	Average Difference
Path 1 (*p* = 1)	−12.9	−16.7	1.2
Path 2 (*p* = 5)	−12.8	−16.3	1
Path 3 (*p* = 10)	−11.8	−14.1	0.6
Path 4 (*p* = 20)	−10.9	−12.9	1.3
Path 5 (*p* = 30)	−10.1	−12.9	1.9
Straight path	1.7	−12.9	7

## Data Availability

Data are contained within the article.
